# Interventions in overcrowding of emergency departments: an overview of systematic reviews

**DOI:** 10.11606/s1518-8787.2020054002342

**Published:** 2020-06-26

**Authors:** Roberto José Bittencourt, Angelo de Medeiros Stevanato, Carolina Thomé N. M. Bragança, Leila Bernarda Donato Gottems, Gisele O’Dwyer

**Affiliations:** I Secretaria de Estado de Saúde do Distrito Federal Fundação de Ensino e Pesquisa em Ciências da Saúde Escola Superior de Ciências da Saúde Distrito Federal Brasil Secretaria de Estado de Saúde do Distrito Federal. Fundação de Ensino e Pesquisa em Ciências da Saúde. Escola Superior de Ciências da Saúde. Distrito Federal, Brasil; II Fundação Oswaldo Cruz Escola Nacional de Saúde Pública Sérgio Arouca Departamento de Administração e Planejamento em Saúde Rio de JaneiroRJ Brasil Fundação Oswaldo Cruz. Escola Nacional de Saúde Pública Sérgio Arouca. Departamento de Administração e Planejamento em Saúde. Rio de Janeiro, RJ, Brasil

**Keywords:** Emergency Service, Hospital, Hospital Administration, Systematic Review

## Abstract

**OBJECTIVE:**

To present an overview of systematic reviews on throughput interventions to solve the overcrowding of emergency departments.

**METHODS:**

Electronic searches for reviews published between 2007 and 2018 were made on PubMed, Cochrane Library, EMBASE, Health Systems Evidence, CINAHL, SciELO, LILACS, Google Scholar and the CAPES periodicals portal. Data of the included studies was extracted into a pre-formatted sheet and their methodological quality was assessed using AMSTAR 2 tool. Eventually, 15 systematic reviews were included for the narrative synthesis.

**RESULTS:**

The interventions were grouped into four categories: (1) strengthening of the triage service; (2) strengthening of the ED’s team; (3) creation of new care zones; (4) change in ED’s work processes. All studies observed positive effect on patient’s length of stay, expect for one, which had positive effect on other indicators. According to AMSTAR 2 criteria, eight revisions were considered of high or moderate methodological quality and seven, low or critically low quality. There was a clear improvement in the quality of the studies, with an improvement in focus and methodology after two decades of systematic studies on the subject.

**CONCLUSIONS:**

Despite some limitations, the evidence presented on this overview can be considered the cutting edge of current scientific knowledge on the topic.

## INTRODUCTION

Overcrowding of Emergency Departments (ED) has become, in recent decades, a critical issue for public health systems worldwide^1^. The demand for emergency care, even in the most developed countries, has grown considerably, due to, among other things, an increase in life expectancy and consequently, a predominance of chronic degenerative diseases, often exacerbated^2^. The countermeasure – an increase in the supply of health services – has not adequately followed this increase in demand and in some health systems, to the contrary, there has been a decrease in the number of hospital beds^3^. Therefore, in view of this excess load, it is difficult for hospital emergency services to fulfill their main mission: to provide care in time and with adequate quality^4,5^.

The invariable scenario of the ED is of completely filled beds, patients placed in hallways, full waiting rooms with patients waiting for care for hours while the staff feels rushed and stressed^6,7^. Consequences are serious: patients who leave the ED before being cared for, dispersion of ambulances and blocked access to medical service, increased length of stay (LOS) in the ED and in the hospital, risk of iatrogenesis, delayed treatment and delayed patient recovery, increased rates of morbidity and mortality, higher operating costs and decreased patient satisfaction^8^.

Though manifested in the ED in a dramatic and inhumane way, overcrowding is a systemic problem interconnected to other difficulties in healthcare^13^. Asplin et al.^14^ devised a theoretical framework that allows for the analysis of overcrowding at the ED using three structural components: input, throughput and output. Input refers to the volume and type of care sought in the ED; output refers to discharge of patients to another care site; and throughput refers to internal ED issues and management^15^.

From this point of view, the overcrowding of ED is a complex systemic process caused by specific bottlenecks, both in ED and in primary care, in the hospital itself and in the health care network as a whole^16^ It is still necessary to establish an integrated model including all three components, allowing us to better understand the full cycle of ED overcrowding.

The objective of this study is to investigate the interventions on the *throughput* component, since the authors consider it the backbone of hospital management and a significant number of systematic reviews on specific interventions of this component were identified. At the same time, this option avoids confusing factors revealed by the two other components, since they present a stronger interface with the other points of the health care system, as revealed by the *overview* of the *input* component described by Van den Heede and Van de Voorde^19^.

Therefore, we carried out an overview of systematic reviews on interventions in the throughput component that sought to solve the ED overcrowding. Based on the available evidence, we hope to list which measures are most effective in dealing with ED overcrowding, providing health managers with an up-to-date view, as well as evaluating the quality of the available literature.

Ethics approval is not applicable for this research.

## METHODS

Guidelines of the Cochrane Collaboration^20^ and Biondi-Zoccai, Umbrella^21^ were followed to carry out an overview of systematic reviews on interventions for ED overcrowding, focusing on the throughput component.

The protocol of this review was recorded on the PROSPERO website (CRD42018087964), on February 7, 2018, available at:

https://www.crd.york.ac.uk/PROSPERO/display_record.php?RecordID=87964.

### Search Strategy

Searches were carried out in the following databases: PubMed, Cochrane Library, EMBASE, Health Systems Evidence, CINAHL, SciELO, LILACS and the CAPES periodicals portal. We also searched for grey literature in Google Scholar. The search strategy used was: ((reduce OR mitigate OR attenuate OR impact OR triage OR intervention) AND (emergency OR emergency room OR emergency department OR ED OR emergency unit) AND (flow OR patient flow OR crowding OR overcrowding) AND (systematic review)). There were no language restrictions in order to obtain as many studies as possible, and publication dates were limited from 2007 to 2018, in order to obtain only studies in a context closer to the current one. Finally, the authors manually searched all reference lists of included studies for other relevant reviews.

### Eligibility Criteria

First, only systematic reviews were included. The criterion established was that, for a study to be considered a systematic review, it must be self-defined as a systematic review. That is, studies defined by the authors as rapid review, integrative review or simply literature review were excluded.

Among the systematic reviews, the ones included were those that analyzed a single intervention on the *throughput* component of ED overcrowding, in order to reduce heterogeneity between the studies. Therefore, studies addressing intervention on *input* or *output*, or more than one intervention on the *throughput* component were not considered.

Effectiveness of intervention was not used as an inclusion criterion. Similarly, the design of the primary studies included in each review was also not a criterion. Reviews about clinical conditions or specific treatments impacting the throughput were excluded. In addition, systematic reviews were only included if there was quantitative data available on some indicator of overcrowding, especially length of stay in the ED, waiting time until being cared for or number of patients who left without treatment.

### Study Selection

According to the search strategy, two authors selected potentially relevant titles and abstracts and the other authors verified their validity. Afterwards, the complete text of the study was obtained, and two authors independently applied the inclusion and exclusion criteria. In case of disagreement, a third author was consulted.

### Data Extraction

Based on the Prisma recommendations^22^, the authors formulated a data extraction spreadsheet on Windows Excel. The items included were: identification of the study (i.e., title, author, year of publication and means of publication); researched data bases; surveyed period; number and design of primary studies included; assessment of the risk of bias of primary studies; population and control group of the studies; interventions; indicators of overcrowding measured; outcomes of intervention; and the conclusions of the author. One author extracted the data from the studies and the others verified the validity. Disagreements were resolved through consensus seeking.

### Quality Assessment

The AMSTAR 2 tool^23^, an updated version of the original AMSTAR, was used to evaluate methodological quality of the reviews. Although the new version is still not as tested and evaluated by other researchers, it was used as it allows for a more detailed and comprehensive evaluation of systematic reviews by including non-randomized studies. AMSTAR 2 is a checklist that considers 16 criteria, 10 from the original AMSTAR with minor changes in the wording, with the addition of six new criteria. Each item is assessed with a “Yes,” “Partially yes” or “No” and hence the methodological quality of the review is classified as “Critically low,” “Low,” “Moderate” or “High.” One author performed the evaluation and another author checked the validity. Disagreements were resolved by seeking consensus with a third reviewer.

## RESULTS

### Search Results

**Figure 1:**
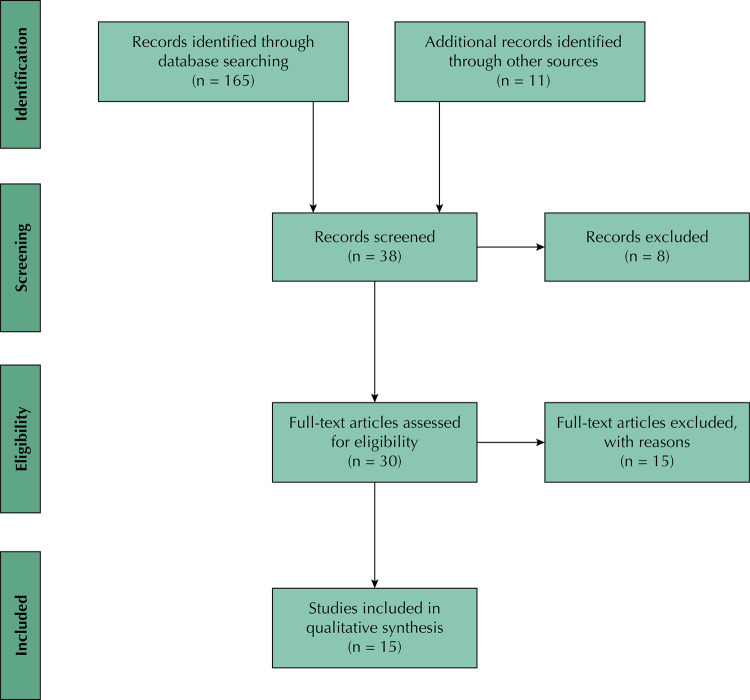
Prisma flow diagram

The electronic database searches yielded 165 results. Another 11 articles were found by manually checking reference lists and searching for grey literature, totaling 176 initial results. After removal of duplicates and articles not deemed eligible, 38 articles were considered potentially relevant and had their abstract screened. Based on their abstract, 30 studies were retrieved for full-text appraisal. After assessment of the entire paper, 15 articles were ultimately included in the on-screen review. The study selection process is shown in the adapted PRISMA flow diagram below (Figure 1).

### Interventions

Fifteen systematic reviews, each with a single intervention, were validated for the study. Their interventions were grouped into four categories: (a) Five studies point to the strengthening of the triage service in the ED; (b) Five deal with the creation of new care zones, such as “Acute Medical Units” or “Rapid Assessment Zones” to treat patients with acute clinical conditions; (c) Three studies analyze the strengthening of the ED team by employing a new professional such as a nurse practitioner or primary care physician; (d) Two studies evaluate new work processes, such as the use of Lean organizational methodology to manage ED and the use of full capacity protocol.

The Box presents the main data of each systematic review. As there is no consensus on which is the best marker for overcrowding^24^, the authors defined length of stay (LOS) in the ED as the main indicator of impact or effectiveness. Only two of the included systematic reviews, by Khangura^24^ and Carter^25^, do not present results on length of stay. In this case, outcomes on other indicators were presented.

**Box:** Summary for characteristics and outcomes of the included studies

**Intervention**	**Author / Country / Year**	**AMSTAR 2**	**Conclusions**	**Number of studies included (N) / Outcomes on LOS**
**Senior Doctor Triage (SDT)** *Placing a senior doctor at the triage in a hospital emergency department (ED)*	Abdulwahid et al.^27^ United Kingdom 2015	Moderate quality	SDT made improvements in the ED overcrowding indicators, especially in the LOS, in most studies.	**N = 25 primary studies** 4 RCT; 2 CCT; 3 Cohorts; 16 Before and After studies. **Results** All studies measured LOS. 3 RCT - Statistically significant reduction in LOS. 16 observational studies - Demonstrated statistically significant LOS reduction.
**Team triage** *The teams were composed by at least one physician and one nurse.*	Thomas **Ming** et al.^28^ China 2016	Moderate quality	In comparison to triage performed by a single nurse, team triage did not provide any statistically significant improvement in LOS or wait times.	**N = 4 primary studies** 4 RCT studies. **Results** 4 studies measured LOS. There was no statistically significant reduction for any patient group.
**Employing a Triage Liaison Physician in the ED** *TLPs establish faster triage flows, depending on the severity or risk of the patient.*	Brian H. **Rowe** et al.^29^ Canada 2011	High quality	The results indicate that a TLP reduces LOS and, to a lesser degree, the number of patients left without being seen in an overcrowded ED. In addition, there was high nurse satisfaction due to increased physician support and high physician satisfaction due to improved quality of work life.	**N = 28 primary studies** 2 RCT; 7 CCT, 2 Cohorts; 1 ITS; 16 Before and After studies. **Results** 19 studies measured LOS. 2 RCT: statistically significant reduction of 37 minutes. 12 not-RCT: statistically significant reductions in the mean LOS between 82 and 11 minutes. 3 not-RCT: unimportant reduction, no change or non-relevant increase in LOS.
**Use of triage nurse ordering (TNO)** *Triage Nurses are authorized to request, from triage, imaging tests, lab tests or ECGs.*	Brian H. **Rowe** et al.^30^ Canada 2011	Moderate Quality	TNO appears to be an effective intervention to reduce LOS, especially in case of patients suspected of having a fracture.	**N = 14 primary studies** 3 RCT; 1 CCT; 2 Cluster-cluster studies; 8 Observational studies. **Results** 14 studies measured LOS. 2 RCT - Significant reduction of 37.2 minutes. 8 not-RCT - Reduction of 51 minutes. When no fracture was suspected: non-significant LOS reduction of 0.93 minutes.
**Comparison of different triage systems:** - Basic triage system versus no formal triage; - Basic triage with variations on team experience or with different triage criteria; - Triage with options management by physician vs. basic triage.	Katherine E. **Harding** et al.^31^ Australia 2011	Critically low quality	Triage systems could have an impact on the flow of patients. There is conflicting evidence on whether basic triage systems can reduce waiting times. The ability to treat cases in triage or to redirect inappropriate visitors improves patient’s LOS.	**N = 25 primary studies** 3 RCT; 5 NRCT; 16 Before and After; 1 ITS. **Results** 7 studies measured LOS. Triage vs. No formal triage: 1 RCT – No significant difference in LOS. Basic triage with different triage criteria: 1 B&A – Mental health triage system, for patients with mental health conditions, caused LOS reduction of 17.5 minutes. Triage with options management by physician vs. basic triage: 5 not-RCT – Reductions in LOS.
**Implementation of Acute Medical Units (AMU)** *A facility in the hospital that acts as the focus for acute medical care for patients who have presented as medical emergencies to hospital.*	Lindsay **Reid** et al.^34^ United Kingdom 2016	Critically low quality	*AMU* is associated with reduced LOS in the hospital, as well as the lower hospital mortality rate. Findings related to other items (patient satisfaction, readmission rate and other outcomes) are inconclusive.	**N = 17 primary studies** 17 observational studies. **Results** 12 studies measured LOS. 12 – Reported reduction of LOS in the hospital. Mean reductions ranged from 0.3 to 2.62 days.
**Implementation of Acute Medical Units (AMU)** *Designated hospital wards to receive medical inpatient presenting with acute medical illness from emergency departments and/or the community.*	Ian **Scott** et al.^35^ Australia 2009	Low quality	The AMUs staffed by multidisciplinary teams led by acute medicine physicians have the potential to improve the quality and the safety of care of a significant proportion of acutely ill medical patients presenting to hospital.	**N = 9 primary studies** 9 Before and After studies. **Results** 4 studies measured LOS. 3 – Statistically significant mean reductions in average LOS in the hospital between 1 and 1.5 days. 1 – Statistically non-significant reduction in the average LOS at the hospital in 0.5 days.
**Implementation of Acute Medical Unit (AMU)** *Designated hospital wards to receive medical inpatient presenting with acute medical illness from emergency departments and/or the community*	L.S. **van Galen** et al.^34^ Netherland 2016	Low quality	The AMU is an effective model to provide acute care and improve efficiency in the care chain thereby improving the patient flow in acute care chain.	**N = 31 primary studies** 27 Before and After; 4 qualitative studies. **Results** 15 studies measured LOS. 3 – Measured LOS at the ED and 12 LOS in the hospital. 3 – Mean hospital LOS reductions, ranging from 0.9 to 1.5 days. 5 – Median hospital LOS reductions, ranging from 0.2 to 2 days, while one study found no difference in median hospital LOS. 1 – Statistically significant reduction of hospital LOS, excluding the time in the ED, from 5.1 to 4.1 days. 1 – Patients who were treated only in the AMU spent less time there on average. 1 – Lower LOS in ED for patients of the Medical Evaluation Unit.
**Implementation of Rapid Assessment Zones** *Areas where patients with acute problems but who are not severely ill and who require limited observation have their investigations started, wait for results and/or receive treatment in a chair or stretcher.*	Michael J **Bullard** et al.^35^ Canada 2012	Moderate quality	The creation of Rapid Assessment Zones appears to be effective, but the available evidence is limited, due to the few studies available.	**N = 4 primary studies** 1 RCT; 1 CCT; 2 Before and After. **Results** 2 studies measured LOS 1 RCT – Statistically non-significant LOS reduction. 1 B&A – Statistically significant LOS reduction. 2 – Analyzed patient subgroups with severity scores of 2 to 5. Both observed LOS reduction for patients with a score of 5.
**Implementation of Short Stay Units (SST)** *The SST is a physical location in a hospital that accommodate patients needing treatments or observation without occupying ED beds or needing to be admitted.*	James **Galipeau** et al.^36^ Canada 2015	High quality	The creation of the Short Stay Units appears to be effective and to save costs, but the available evidence is limited and inconclusive.	**N = 5 primary studies** 5 RCT studies. **Results** 4 studies measured LOS. 2 – Statistically significant reduction in median LOS. 1 – Statistically non-significant reduction in median LOS. 1 – Statistically significant reduction in mean LOS.
**Employing general practitioners (GPs) in ED to care for patients with non-urgent health problems**	Jaspreet K **Khangura** et al.^25^ United Kingdom 2014	Moderate Quality	There is very weak evidence to suggest that GPs may use less resources to treat non-urgent patients in ED. Thus, while the intervention may provide cost-savings, it is unclear if less resource utilization translates into improved outcomes.	**N = 3 primary studies** 3 NRCT studies. **Results** Zero studies measured LOS. Other outcomes: Reduction of laboratory and image diagnostic tests requested. Reduction in the number of hospitalizations. Rates of return to the ED or basic care in 30 days did not change. Cost reduction. Satisfaction of patients with care did not change.
**Employing nurse practitioners (NPs) in the ED** *NPs may evaluate, diagnose and treat patients, as well as refer them to other specialties and prescribe medication.*	Natasha **Jennings** et al.^37^ Australia 2015	Moderate quality	NPs’ services on the ED have a positive impact on patient’s satisfaction and waiting times. There was no difference in cost-effectiveness between NP and other professionals. The quality of ED care with NPs was important with respect to patient safety.	**N = 14 primary studies** 2 RCT; 2 NRCT; 10 observational studies. **Results** 9 studies measured LOS. 5 – Statistically relevant difference in LOS, ranging from 6 to 76 min. 4 – No statistically significant difference in the LOS.
**Employing nurse practitioners (NPs) in the ED** *NPs may evaluate, diagnose and treat patients, as well as refer them to other specialties and prescribe medication.*	Alix J.E. **Carter**^26^ USA 2007	Low quality	The results suggest that the addition of a staff member dedicated to seeing minor treatment patients will improve wait times and improve patient satisfaction, with little or no impact on quality of care.	**N = 37 primary studies** 3 RCT; 18 Case-control studies; 8 Cohorts; 8 Surveys. **Results** Zero studies measured LOS. Other outcomes: Statistically significant difference in favor of NPs in relation to cost – effectiveness. Positive difference in relation to LOS in the ED, but without statistical treatment.
**Use of the Full Capacity Protocol (FCP)** *Create a designated area in the hospital to admit patients from ED who need hospitalization, so they do not wait in the ED itself. The triggers for FCP utilization are: use of stretchers in the ED and waiting time for hospitalization.*	Cristina **Villa-Roel** et al.^38^ Canada 2012	Low quality	As the evidence on FCP implementation to solve overcrowding is limited, there are few studies available and when available, without statistical analysis.	**N = 5 primary studies** 1 RCT; 3 Before and After; 1 ITS. **Results** 1 study measured LOS. 1 B&A – LOS at the ED had an average reduction of 5 hours. Subgroup analysis: For clinical patients, there was mean ED LOS reduction of 9 hours, for surgical patients, of 1.6 hours; for mental health patients, of 9.2 hours. LOS at the hospital had mean reduction of 1 day for clinical patients; 0.8 for surgical patients; 0.8 day for mental health patients.
**Use of the Lean Thinking approach for re-designing ED processes**	M **Tanzariello** et al.^40^ Italy 2015		Managing hospitals through Lean becomes a prerogative of an excellent patient-oriented healthcare system: it seems to be critical for a better value-based healthcare.	**N = 9 primary studies** All studies are Before and After. **Results** 7 studies measured LOS. 6 - Reduction of LOS. 1 - Both reduction and increase of LOS, depending on where the study was conducted.

Caption: AMU: acute medical unit; B&A: before and after; CCT: clinical controlled trial; ED: emergency department; FCP: full capacity protocol; GP: general practitioner; ITS: interrupted time series; LOS: length of stay; NP: nurse practitioner; NRCT: non-randomized controlled trial; RAZ: rapid assessment zone; RCT: randomized controlled trial; TLP: triage liaison physician; TNO: triage nurse ordering; SSU: short stay unit.

### Quality of the Studies by the AMSTAR 2 Standard

Of the included studies, two followed closely the AMSTAR 2 guidelines and have high methodological quality, six reviews have medium quality, four reviews have low quality and three have critically low methodological quality. Therefore, more than half of the reviews (8 out of 15) are of high or moderate quality.

**Figure 3:**
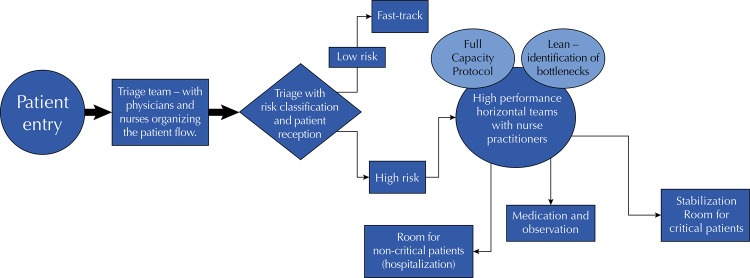
Interventions identified in all the studies

Item 5 received the highest amount of “yes” marks and concerns the performance of study selection in duplicate. In addition, items 13, 14 and 16 were largely adopted, reflecting that authors observed possible risk of bias, heterogeneity in the results and conflicts of interest. Item 10, on the other hand, which evaluates whether the authors informed the funding sources of the included primary studies did not receive a single positive mark among all reviews. Items 2 and 7 (respectively concerning defining and publishing an *a priori* protocol and making a list of excluded studies available) were also not well-evaluated in general.

**Figure 2:**
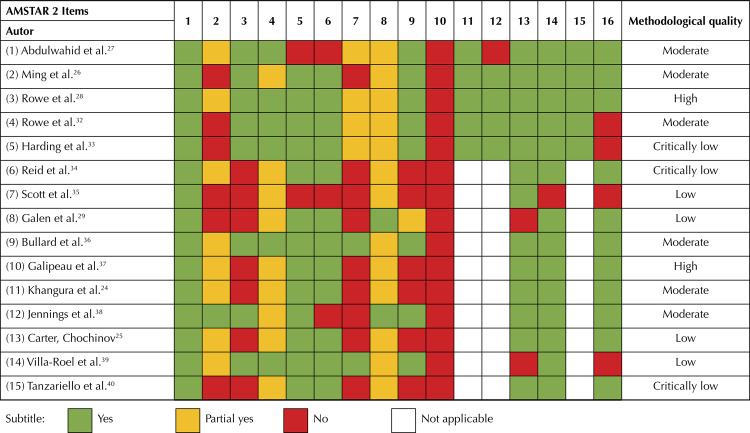
AMSTAR 2 assessment results for each included review.

A more complete analysis of the AMSTAR 2 assessment is presented in Figure 2, with identification of each item and a box summarizing the results.

## DISCUSSION

Emergency department overcrowding is caused by many complex causes in a nonlinear cause-and-effect relationship. Therefore, it becomes a difficult problem to solve, in which multiple interventions are required to try to solve or even mitigate it. Yet, the results presented show that intervening exclusively on the ED throughput – even if changes are not concurrently made to primary care or hospital management – is already enough to reduce ED overcrowding, at least in part. Thus, the purpose of this study is not to suggest a perfect solution, but to show, based on current evidence, which measures intrinsic to the ED are effective in alleviating overcrowding.

The use of physicians in triage was the most successful intervention. Four reviews addressed such a solution, all being of moderate quality. Two of them brought 19 primary studies each, and the other one included only four studies, but all were randomized controlled trials (RCT). The evidence was therefore considered the most robust among those found. In the studies, either the physician acted alone or in conjunction with a nurse, allowing diagnostic procedures and treatments to begin even before the patient was formally treated. Length of stay and waiting time per patient per physician were reduced in most cases. Despite these findings, Ming^26^ argues that team triage has no clear benefit and Abdulwahid^27^ draws attention to insufficient evidence. In short, following Rowe^28^, the doctor’s job in triage should not be the main goal in the search for a solution to the problem, but it is a measure that yields good results. The review on the use of a primary care physician in the ED is of moderate AMSTAR 2 quality. Since there are few primary studies (n = 3) available and no overcrowding indicators such as length of stay or waiting time for care have been addressed, it is difficult to judge the true potential of intervention on overcrowded conditions. It is also necessary to consider the profile of the patients who visit a particular ED. In case of high amounts of patients with low severity issues, this intervention tends to work better.

Another intervention on triage that has shown promise is allowing triage nurses to request diagnostic tests, even before a doctor sees the patient. This intervention depends, to some extent, on the profile of patients who usually show up at the given emergency department. Still, it is a valid option, especially for patients with suspected fractures. It should also be noted that nurses must undergo training before performing such a role.

Employing nurse practitioners in ED was evaluated in two reviews, one of moderate quality and one of low quality. However, there are variations among the primary studies in the functions performed by these professionals. Normally, they may evaluate, diagnose and treat patients, refer them to other specialties and prescribe medications, all according to defined protocols. Conflicting evidence was found on the impact of nurse practicioners on LOS, with a slight downward trend. The indicator that showed the greatest impact was patient satisfaction, but the item was evaluated in few primary studies.

Implementing “Acute Medical Units” is also a well-studied and effective solution. The model and concept of “Acute Medical Units” may vary somewhat between studies, but they are all designated areas for patients with acute clinical conditions without immediate risk of death, but that need to remain under surveillance while receiving immediate care (e.g. patients with headaches, severe chest pain or patients with decreased level of consciousness). The terms given to the structure in the reviews are Medical Assessment Unit, Acute Medical Unit, Short-Stay Unit and Rapid Assessment Zones. Five reviews address this intervention. However, three reviews are of low quality and one is of critically low quality. One review is of high quality and includes only randomized controlled trials, which enhances evidence. This review noted mainly a reduction in LOS and costs. The others also consistently show a reduction in LOS, as well as other outcomes, such as reduction in the number of repeated visits to the ED, decrease in the mortality rate, and increase in patient satisfaction. The conclusion is that that the implementation of “Acute Medical Units” (or similar areas) is an intervention that generates a reduction in length of stay and can be used safely.

The intervention on the use of a Full Capacity Protocol is found in only one review, which included only one before-after study addressing solely the FCP, and four other studies of multiple interventions with an FCP component. Hence, this review provides incomplete findings that does not allow conclusions to be drawn. In any case, outcomes on length of stay were positive.

The systematic review using the Lean Thinking approach for re-designing ED processes has only few primary studies included (n = 9) and is of low quality, according to the AMSTAR 2 standards. Nevertheless, given the recent expansion of the Lean method towards health management, especially when used to identify potential bottlenecks in ED, the authors of this overview believe it is an effective intervention, if properly applied by trained employees. The results of the systematic review^29^, in general, corroborate this thesis: in the majority of cases, there was a decrease in length of stay and waiting time, as well as a reduction in the cost per patient and in the number of patients who left the ED before being treated. There was also an increase in patient satisfaction.

The following figure summarizes the interventions that were identified in all the studies.

De Freitas et al.^30^carried out, in 2018, an overview of interventions that could improve the flow of patients in the ED, and found 13 systematic reviews, with 22 interventions grouped as follows: (1) diagnostic services; (2) short-duration units; (3) interventions led by nurses; (4) physician-directed interventions; (5) changes in work processes and (6) miscellaneous changes. However, the methodology used differed from the present research in a fundamental aspect - studies with multiple interventions were included. In this way, the confounding factors are amplified and, according to the authors, the evidence of that overview were of low quality.

### Limitations

In eleven years of studies on the subject, there is a clear evolution on evidence related to interventions that positively impact ED overcrowding. The 2007 systematic review by Bittencourt and Hortale^31^ highlights 21 distinct interventions with positive interference on the length of stay of patients in ED, but does not include any randomized controlled trials. In this 2018 overview, larger equilibrium was observed between observational studies and randomized controlled trials, although the former still prevailed. In addition, most of the 15 systematic reviews analyzed the possibility of bias in the primary studies and five of them defined quantitative methods for statistical analysis, revealing significance of the data found.

However, differences remain on data reports and/or indicators of effectiveness in studies, making it difficult to compare them and requiring rigor in the elaboration of a synthetic narrative. In a certain way, these limitations were minimized to the extent that only studies that evaluated the effectiveness of a single intervention were selected.

Another important factor to be considered is that the primary studies were conducted, in their majority, in developed countries. Health conditions in a country directly influence the volume and types of demand for emergency services. Developed countries probably rely on better-structured and better-funded ED to overcome overcrowding. Therefore, the results presented were tested in developed countries and may not manifest in the same manner in underdeveloped or developing countries.

## CONCLUSION

This overview consisted of 15 systematic reviews that bring four possible types of interventions into the ED throughput component, with positive results on a variety of overcrowding indicators, especially patients’ length of stay in the ED.

Considering the quantity and quality of systematic reviews, proven effective interventions are: (1) the use of a physician/nurse to perform and supervise triage and flow of patients; (2) strengthening the care team through the use of nurse practitioners; (3) implementation of new areas for caring of patients with acute non-critical conditions or areas to medicate and observe patients before assessing severity and (4) use of the Lean methodology and full capacity protocols.

It can be inferred that this study an overview of the available literature, by virtue of being a third-generation research, obtained a consistent degree of evidence. The prevalence of high and moderate quality reviews allows the recommendation of the evidence presented as the cutting edge of current scientific knowledge. Already identified limitations notwithstanding, the studies may be used if the tacitly acquired local knowledge is considered.

It has also been shown that the concept of overcrowding in emergency departments is a phenomenon that essentially reveals an imbalance between demand and supply of health services. These are fundamentally related to a growing lack of public-sector hospital beds; low integration between health care networks, which hamper an integrated healthcare system, and low effectiveness of basic care services. Nevertheless, ED overcrowding is not the only manifestation of this demand-supply imbalance: queues and other unmet healthcare needs of various services are increasingly found. This issue will remain serious, as healthcare systems are under serious social and economic constraints, thus imposing severe budget restrictions to public healthcare.

## References

[1] 1. Pines JM, Hilton JA, Weber EJ, Alemade AJ, Al Shabanah H, Anderson PD, et al. International perspectives on emergency department crowding. Acad Emerg Med. 2018(12):1358-70. 10.1111/j.1553-2712.2011.01235.x 22168200

[2] 2. Berchet C. Emergency care services: trends, drivers and interventions to manage the demand. Paris: Organisation for Economic Cooperation and Development (OECD); 2015.

[3] 3. Hsia RY, Kellermann AL, Shen YC. Factors associated with closures of emergency departments in the United States. JAMA. 2011;305(19):1978-85. 10.1001/jama.2011.620 PMC4063529 21586713

[4] 4. Fatovich DM, Hirsch RL. Entry overload, emergency department overcrowding, and ambulance bypass. Emerg Med J. 2003;20(5):406-9. 10.1136/emj.20.5.406 PMC1726189 12954675

[5] 5. American College of Emergency Physicians -ACEP. Policy statements. Ann Emerg Med. 2013;61(6):602-3. 10.1016/j.annemergmed.2013.02.012

[6] 6. Derlet R, Richards J, Kravitz R. Frequent overcrowding in U.S. emergency departments. Acad Emerg Med. 2001;8(2):151-5. 10.1111/j.1553-2712.2001.tb01280.x 11157291

[7] 7. Pines JM. Moving closer to an operational definition for ED crowding. [letter]. Acad Emerg Med. 2007;14:382-3. 10.1197/j.aem.2006.11.018 17401000

[8] 8. Sun BC, Hsia RY, Weiss RE, Zingmond D, Liang LJ, Han W, et al. Effect of emergency department crowding on outcomes of admitted patients. Ann Emerg Med. 2013;61(6):605-11e6. 10.1016/j.annemergmed.2012.10.026 PMC3690784 23218508

[9] 9. Johnson KD, Winkelman C. The e ffect of e mergency department crowding on patient outcomes. Adv Emerg Nurs J. 2011;33(1):39-54. 10.1097/TME.0b013e318207e86a 21317697

[10] 10. Krochmal P, Riley TA. Increased health care costs associated with ED overcrowding. Am J Emerg Med. 1994;12(3):265-6. 10.1016/0735-6757(94)90135-x 8179727

[11] 11. Salway RJ, Valenzuela R, Shoenberger JM, Mallon WK, Viccellio A. Emergency department (ED) overcrowding: evidence-based answers to frequently asked questions. Rev Med Clin Las Condes. 2017;28(2):213-9. 10.1016/j.rmclc.2017.04.008

[12] 12. Morley C, Unwin M, Peterson GM, Stankovich J, Kinsman L. Emergency department crowding: a systematic review of causes, consequences and solutions. PLoS One. 2018;13(8):e0203316. 10.1371/journal.pone.0203316 PMC6117060 30161242

[13] 13. Jeanmonod D, Jeanmonod R. Overcrowding in the emergency department and patient safety. In: Firstenberg MS, Stawicki SP, editors. Vignettes in patient safety. Vol, 2. London (UK): IntechOpen; 2018. p. 57-72. 10.5772/intechopen.69243

[14] 14. Asplin BR, Magid DJ, Rhodes KV, Solberg LI, Lurie N, Camargo JR CA. A conceptual model of emergency department crowding. Ann Emerg Med. 2003;42(2):173-80. 10.1067/mem.2003.302 12883504

[15] 15. Moskop JC, Sklar DP, Geiderman JM, Schears RM, Bookman KJ. Emergency department crowding, part 1 - concept, causes, and moral consequences. Ann Emerg Med. 2009;53(5):605-11. 10.1016/j.annemergmed.2008.09.019 19027193

[16] 16. Asaro PV, Lewis LM, Boxerman SB. The impact of input and output factors on emergency department throughput. Acad Emerg Med. 2007;14(3):235-42. 10.1197/j.aem.2006.10.104 17284466

[17] 17. Doupe MB, Chateau D, Chochinov A, Weber E, Enns JE, Derksen S, et al. Comparing the effect of throughput and output factors on emergency department crowding: a retrospective observational cohort study. Ann Emerg Med. 2018;72(4):410-9. 10.1016/j.annemergmed.2018.04.001 29804715

[18] 18. Grumbach K, Keane D, Bindman A. Primary care and public emergency department overcrowding. Am J Public Health. 1993;83(3):372-8. 10.2105/ajph.83.3.372 PMC1694659 8438975

[19] 19. Van den Heede K, Van de Voorde C. Interventions to reduce emergency department utilisation: a review of reviews. Health Policy. 2016;120(12):1337-49. 10.1016/j.healthpol.2016.10.002 27855964

[20] 20. Becker LA, Oxman AD. Overviews of reviews. In: Higgins JPT, Green S, editors. Cochrane handbook for systematic reviews of interventions. Chichester (UK): John Wiley; 2008. p. 607-31.

[21] 21. Biondi-Zoccai G, editor. Umbrella reviews : evidence synthesis with overviews of reviews and meta-epidemiologic studies. New York: Springer International; 2016.

[22] 22. Moher D, Liberati A, Tetzlaff J, Altman DG; PRISMA Group. Preferred Reporting Items for Systematic Reviews and Meta-Analyses: The PRISMA statement. PLoS Med. 2009;6(7):e1000097. 10.1371/journal.pmed.1000097 PMC2707599 19621072

[23] 23. Shea BJ, Reeves BC, Wells G, Thuku M, Hamel C, Moran J, et al. AMSTAR 2: a critical appraisal tool for systematic reviews that include randomised or non-randomised studies of healthcare interventions, or both. BMJ. 2017;358:j4008. 10.1136/bmj.j4008 PMC5833365 28935701

[24] 24. Khangura JK, Flodgren G, Perera R, Rowe BH, Shepperd S. Primary care professionals providing non-urgent care in hospital emergency departments. Cochrane Database Syst Rev. 2012;11:CD002097. 10.1002/14651858.CD002097.pub3 PMC4164956 23152213

[25] 25. Carter AJE, Chochinov AH. A systematic review of the impact of nurse practitioners on cost, quality of care, satisfaction and wait times in the emergency department. CJEM. 2007;9(4):286-95. 10.1017/s1481803500015189 17626694

[26] 26. Ming T, Lai A, Lau PM. Can team triage improve patient flow in the emergency department? A systematic review and meta-analysis. Adv Emerg Nurs J. 2016;38(3):233-50. 10.1097/TME.0000000000000113 27482995

[27] 27. Abdulwahid MA, Booth A, Kuczawski M, Mason SM. The impact of senior doctor assessment at triage on emergency department performance measures: systematic review and meta-analysis of comparative studies. Emerg Med J. 2016;33(7):504-13. 10.1136/emermed-2014-204388 26183598

[28] 28. Rowe BH, Guo X, Villa-Roel C, Schull M, Holroyd B, Bullard M, et al. The role of triage liaison physicians on mitigating overcrowding in emergency departments: a systematic review. Acad Emerg Med. 2011;18(2):111-20. 10.1111/j.1553-2712.2010.00984.x 21314769

[29] 29. Galen LSV, Lammers EMJ, Schoonmade LJ, Alam N, Kramer MHH, Nanayakkara PWB. Acute medical units: the way to go? A literature review. Eur J Intern Med. 2017;39:24-31. 10.1016/j.ejim.2016.11.001 27843036

[30] 30. De Freitas L, Goodacre S, O’Hara R, Thokala P, Hariharan S. Interventions to improve patient flow in emergency departments: an umbrella review. Emerg Med J. 2018;35(10):626-37. 10.1136/emermed-2017-207263 30093379

[31] 31. Bittencourt RJ, Hortale VA. Intervenções para solucionar a superlotação nos serviços de emergência hospitalar: uma revisão sistemática. Cad Saude Publica. 2009;25(7):1439-54. 10.1590/S0102-311X2009000700002 19578565

[32] 32. Rowe BH, Villa-Roel C, Guo X, Bullard M, Ospina M, Vandermeer B, et al. The role of triage nurse ordering on mitigating overcrowding in emergency departments: a systematic review. Acad Emerg Med. 2011;18(12):1349-57. 10.1111/j.1553-2712.2011.01081.x 21692901

[33] 33. Harding KE, Taylor NF, Leggat SG. Do triage systems in healthcare improve patient flow? A systematic review of the literature. Aust Health Rev. 2011;35(3):371-83. 10.1071/AH10927 21871201

[34] 34. Reid LEM, Dinesen LC, Jones MC, Morrison ZJ, Weir CJ, Lone NI. The effectiveness and variation of acute medical units: a systematic review. Int J Qual Health Care. 2016;28(4):433-46. 10.1093/intqhc/mzw056 27313174

[35] 35. Scott I, Vaughan L, Bell D. Effectiveness of acute medical units in hospitals: a systematic review. Int J Qual Health Care. 2009;21(6):397-407. 10.1093/intqhc/mzp045 19903756

[36] 36. Bullard MJ, Villa-Roel C, Guo X, Holroyd BR, Innes G, Schull MJ, et al. The role of a rapid assessment zone/pod on reducing overcrowding in emergency departments: a systematic review. Emerg Med J. 2011;29(5):372-8. 10.1136/emj.2010.103598 21515880

[37] 37. Galipeau J, Pussegoda K, Stevens A, Brehaut JC, Curran J, Forster AJ, et al. Effectiveness and safety of short-stay units in the emergency department: a systematic review. Acad Emerg Med. 2015;22(8):893-907. 10.1111/acem.12730 26201285

[38] 38. Jennings N, Clifford S, Fox AR, O’Connell J, Gardner G. The impact of nurse practitioner services on cost, quality of care, satisfaction and waiting times in the emergency department: a systematic review. Int J Nurs Stud. 2015;52(1):421-35. 10.1016/j.ijnurstu.2014.07.006 25443302

[39] 39. Villa-Roel C, Guo X, Holroyd B, Innes G, Wong L, Ospina M, et al. The role of full capacity protocols on mitigating overcrowding in EDs. Am J Emerg Med. 2012;30(3):412-20. 10.1016/j.ajem.2010.12.035 21367554

[40] 40. Tanzariello M, Marventano S, Bucci S, De Leva AC, Ricciardi W, Belvis AG. Emergency department crowding and access block: is lean thinking a smart answer? A systematic review: Stephano Marventano. Eur J Public Health. 2015;25 Suppl 3:ckv175.145. 10.1093/eurpub/ckv175.145

